# Compound Heat Vulnerability in the Record-Breaking Hot Summer of 2022 over the Yangtze River Delta Region

**DOI:** 10.3390/ijerph20085539

**Published:** 2023-04-17

**Authors:** Shaojing Jiang

**Affiliations:** 1 Department of Geography and Spatial Information Techniques, Ningbo University, Ningbo 315211, China; jiangshaojing@mail.bnu.edu.cn; 2 State Key Laboratory of Earth Surface Processes and Resource Ecology, College of Global Change and Earth System Science, Beijing Normal University, Beijing 100875, China

**Keywords:** heat vulnerability, heat risk, Yangtze River Delta, heat waves, heat adaptation

## Abstract

Hourly meteorological data and multisource socioeconomic data collected in the Yangtze River Delta (YRD) region were used to analyze its heat vulnerability during the record-breaking hot summer of 2022 in both daytime and nighttime. Over forty consecutive days, daytime temperatures exceeded 40 °C, and 58.4% of the YRD region experienced 400 h with temperatures hotter than 26 °C during the nighttime. Only 7.5% of the YRD region was under low heat risk during both daytime and nighttime. Strong heat risk combined with strong heat sensitivity and weak heat adaptability led to strong heat vulnerability during both daytime and nighttime in most areas (72.6%). Inhomogeneity in heat sensitivity and heat adaptability further aggravated the heterogeneity of heat vulnerability, leading to compound heat vulnerability in most regions. The ratios of heat-vulnerable areas generated by multiple causes were 67.7% and 79.3% during daytime and nighttime, respectively. For Zhejiang and Shanghai, projects designed to decrease the urban heat island effect and lower the local heat sensitivity are most important. For Jiangsu and Anhui, measures aiming to decrease the urban heat island effect and improve heat adaptability are most important. It is urgent to take efficient measures to address heat vulnerability during both daytime and nighttime.

## 1. Introduction

The Yangtze River Delta (YRD) region accounts for only 2.2% of the land area of China, but comprises 16% of the national population and 24.5% of the national economic aggregate of China; thus, this region has become an important carrier and support for national economic development [1]. Heat waves are among the meteorological disasters causing the highest mortality rates in the YRD urban agglomeration each summer [2,3]; heat waves not only seriously threaten the lives and health of residents [4,5,6,7], but also introduce vast economic losses [8,9]. Under the background of global warming and continuous urbanization [10,11], the frequency and intensity of summer heat waves are continuously increasing [12]. In the future, the YRD region will face more frequent, more severe, and longer-lasting summer heat waves. It is thus urgent to take effective measures to address the vulnerability caused by summer heat waves [13].

The vulnerability of a population to summer heat is characterized by the degree to which urban systems are affected by heat [14]. During a heat wave, the biophysical and social complexity of urban systems will affect the population’s vulnerability [15]. The heat risk, heat sensitivity, and heat adaptability are major concerns affecting the heat vulnerability, and these factors have been studied in many research studies [16]. Regarding the heat risk, daytime heat indicators, e.g., daily maximum temperature (*T_max_*), and the number of hot days are often the main factors considered. However, recent studies have indicated that an increase in summer nighttime heat interferes with normal sleep, which leads to impaired immune systems, impaired mental health, increased risks of cardiovascular disease, chronic disease, inflammation, etc. Researchers have populated that increased nighttime heat in summer could increase global mortality by 60% by the end of the century [17,18]. However, few studies have considered nighttime heat vulnerability due to the lack of nighttime temperature observations, especially hourly observations.

Sensitivity and adaptability are important indicators in heat vulnerability assessments. Studies have shown that population density, age distribution, the level of unemployment, and the amount of education are significantly correlated with heat-related diseases and mortality [19]. The income level and the distribution of medical facilities and green spaces are also significantly correlated with heat adaptability [20]. When heat adaptability is poor, there are increased property losses and damages to livelihoods, and decreased health [21]. To resist worsening summer heat waves, developed countries have strengthened relevant treatment projects by mapping heat vulnerability to ensure priority protection for residents in high-risk areas [22], and these government (or policy) initiatives have achieved promising results [23,24,25].

Although China has begun to realize the importance of management addressing summer heat waves, the official response to disasters resulting from heat waves is slow in comparison to the response to other disasters, such as droughts, floods and typhoons [26,27,28]. In contrast to Western developed countries, few studies have explored the anticipation and mitigation of the effects of heat waves by drawing finer-resolution vulnerability maps in China [29]. Developing a coherent response to heat waves is hampered as urbanization and the development of more urban centers increase [30,31]; this is especially true for the YRD region [32].

The YRD region experienced record-breaking summer heat in 2022, thus providing us with a good opportunity to evaluate heat vulnerability. Therefore, in this work, multisource data, including hourly meteorological observations collected during both daytime and nighttime, population data, and other kinds of socioeconomic data were collected and a vulnerability assessment framework was constructed. Afterward, the heat vulnerability distribution and the dominant causes of heat vulnerability over the YRD region were analyzed. This study can provide theoretical support and a reference for future studies involving heat vulnerability assessments. It can also provide a scientific basis for the government to form targeted measures to reduce damage in heat-vulnerable areas according to their domain-specific causes and promote the construction of ecologically livable cities in the YRD region. This is especially critical as climate warming continues.

## 2. Materials and Methods

**Figure 1:**
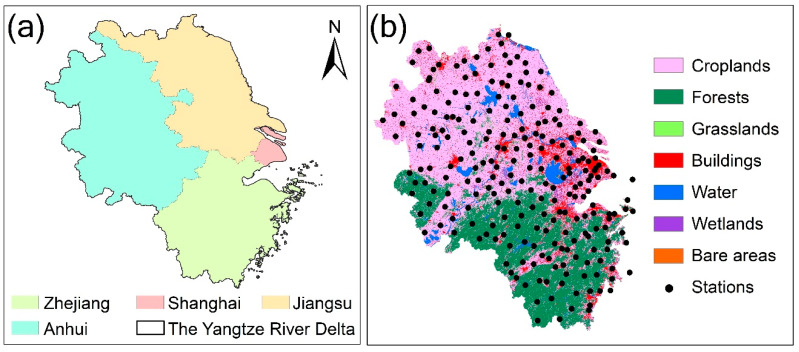
Administrative divisions within the study area (**a**), the locations of 220 meteorological stations (**b**) and the land surface type classifications (**b**) in the YRD region with a resolution of 30 m.

The YRD region is located in the downstream region of the Yangtze River basin in China, covering three provinces and one megacity, i.e., Zhejiang Province, Jiangsu Province, Anhui Province, and Shanghai city (Figure 1a). Ground-based hourly meteorological air temperature (*T_a_*) observations collected at 220 stations and hourly gridded *T_a_* data with a resolution of 7 × 7 km^2^ derived from multiple ground-observation and satellite-observation sources from July to August 2022 (Figure 1b) were obtained from the Chinese Meteorological Administration. The quality-control procedures applied herein included the plausible value, temporal consistency, internal consistency, historical value, and spatial distribution checks already performed by the China Meteorological Administration. These quality control procedures conducted that station *T_a_* data had fewer than 0.01% errors and missing data were less than 0.05%. The gridded *T_a_* data were also of high quality, with deviations less than −0.13 °C.

Land cover data in 2020, with a resolution of 30 m (Figure 1b), were collected from the Resource and Environment Science Data Center (https://www.resdc.cn/Default.aspx, accessed on 1 August 2022). Population density data in 2020, with a resolution of 1 km^2^, were collected from the U.S. Department of Energy’s Oak Ridge National Laboratory (https://landscan.ornl.gov/, accessed on 1 August 2022). The locations of hospitals in 2022 were collected from the Amap (http://ditu.amap.com/, accessed on 1 August 2022). Additionally, socioeconomic data for 2021 were collected from the National Bureau of Statistics (http://www.stats.gov.cn/tjsj/, accessed on 1 September 2022). These data included the distributions of the child population, elderly population, unemployed population, uneducated population, consumption ability levels, medicine consumption levels, and well-educated population in each district. All of the abovementioned data were used to analyze both heat sensitivity and heat adaptability.

In this study, I used the China Meteorological Administration’s definitions of a hot day (*T_max_* ≥ 35 °C) and a hot night (daily minimum temperature (*T_min_*) ≥ 26 °C), which would make people feel uncomfortable during daytime work or nighttime sleep. The hours of 8:00–20:00 (Beijing time) and 20:00–8:00 (Beijing time) were considered daytime and nighttime in this work because these are typically the regular hours when people are expected to work or to be asleep [33]. Meanwhile, all of the observed *T_min_* occurred in the hours of 20:00–8:00 over the YRD region in the summer of 2022, with 93.5% of the observed *T_min_* occurring in the early morning (4:00–6:00) [34]. Additionally, all of the observed *T_max_* occurred in the hours of 8:00–20:00 over the YRD region in the summer of 2022, with 91.9% of the observed *T_max_* occurring in the afternoon (13:00–16:00). Therefore, hot days, hot nights, hot daytime hours, and hot nighttime hours (see Table 1 for details) were defined to calculate heat indicators during daytime and nighttime, which were derived from the daily and hourly *T_a_* data and considered when assessing the heat risk index (see Table 2 for details).

The heat sensitivity index and heat adaptation index were evaluated based on socioeconomic datasets (see Table 2 for details). The gridded population density data were divided into 1000 individual levels to represent the population density index. This classification was also applied to other population-related indicators, including ‘Pop’, ‘Child’, ‘Elder’, ‘Jobless’, ‘Uneducated’, and ‘Well-educated’ (see Table 2 for details). The area with a higher sensitivity tends to have a more dense population, more young children and older people, more unemployed people, and uneducated people. A spatial buffer analysis and spatial overlap analysis were performed for each hospital location point. Twelve kilometers was selected as the radius in the spatial analysis according to the health care coverage area. The medical levels were divided into three categories according to the local hospital levels. To apply the spatial heat vulnerability calculation, the resolution of all indicators was unified to 7 × 7 km^2^ after the data quality-assurance procedure was completed. The dominant causes of heat vulnerability were divided into seven categories related to the grid-scale assessments of the heat risk, heat sensitivity, and heat adaptability (see Table 3 for details).

The daytime/nighttime heat risk, heat sensitivity, and heat adaptability indexes were calculated based on the following equation:

(1) $$IND=\sum_{1}^{n}\left[{(D}_{i}{\times W}_{i}\right)]$$

where *IND* represents the index of daytime heat risk or nighttime heat risk or heat sensitivity or heat adaptability, *D_i_* represents the *i*-th indicator (see Table 2 for details), *W_i_* represents the weight coefficient of the *i*-th indicator, and *n* represents the total number of indicators used to evaluate *IND*. To reduce the magnitude differences among the different indicators, the entropy weights method was employed to obtain each *W_i_*, which has been widely used in comprehensive evaluation studies that use different evaluation indexes [35,36,37]. In the entropy weights method, the weights of different indexes are determined according to the degree of dispersion and the detailed equations could be found in the related works [38].

Additionally, a normalization procedure (Equation (2)) was applied to each indicator and each *IND* result to unify different dimensions in order to remove the impact of differences in units or magnitude of different indicators:

*Y*_(*i*,*j*)_= [*X*_(*i*,*j*)_ − *min*(*X*_*j*_)]/[*max*(*X*_*j*_) − *min*(*X*_*j*_)] (2)

where *X*_(*i*,*j*)_ and *Y*_(*i*,*j*)_ represent the value of the *i*-th pixel of the *j*-th indicator before and after normalization processing, respectively, *X_j_* represents the data sequence of the *j*-th indicator, and *max* and *min* represent the minimum and maximum values of the function, respectively.

Based on the evaluation results of the heat risk, heat sensitivity, and heat adaptability indexes, the daytime/nighttime heat vulnerability index was calculated using the following equation:

*VI* = *EI* × *W*_*EI*_ + *SI* × *W*_*SI*_ − *ADI* × *W*_*ADI*_ (3)

where *EI*, *SI*, *ADI*, and *VI* represent the daytime/nighttime heat risk, heat sensitivity, heat adaptability, and daytime/nighttime heat vulnerability indexes, respectively, and *W_EI_*, *W_SI_*, and *W_ADI_* represent the weight coefficients of *EI*, *SI*, and *ADI*, respectively. According to the EWM method performed above, *W_EI_*, *W_SI_*, and *W_ADI_* were 0.3319, 0.3528, and 0.3153, respectively. Weight coefficients were consistent during daytime and nighttime to better compare their heat vulnerability since same *SI* and *ADI* were applied in calculating the daytime and nighttime *VI*. The normalization procedure was applied to the *VI* after this calculation.

## 3. Results

### 3.1. Strong Heat Intensity and Heat Frequency in the YRD Region during Both Daytime and Nighttime

**Figure 2:**
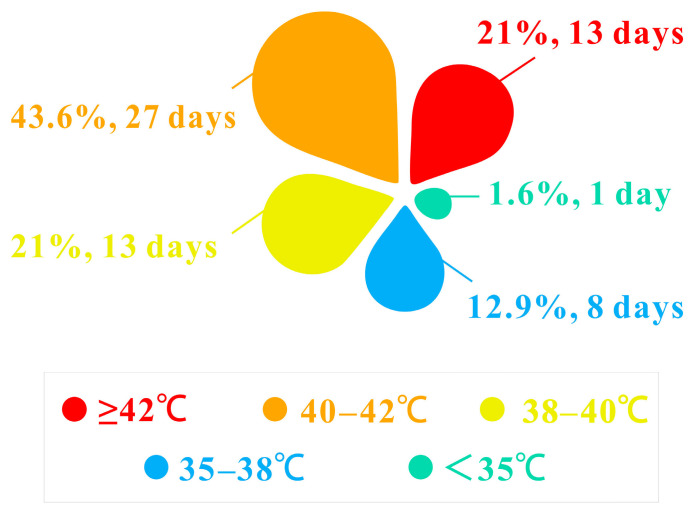
Distributions of *T_max_* in the YRD region from July to August 2022.

In July of 2022, the YRD region had entered a “roasting” mode followed by a record-breaking summer of extreme heat. For July and August 2022, there were 61 days when *T_max_* ≥ 35 °C! For 40 days, *T_max_* ≥ 40 °C (Figure 2)! For the 61 days, the average *T_max_* value reached 39.1 ± 1.7 °C, while the average *T_min_* value reached 29.0 ± 1.9 °C. The total number of days when the regional *T_max_* value ranged from 40 to 42 °C was 27 days (Figure 2). During daytime on hot days, the total number of hours when *T_a_* exceeded 35 °C reached 9 ± 1.0 h, whereas at night, the duration over which *T_a_* exceeded 26 °C reached 12 h. These results reveal the very severe heat situation that occurred over the YRD region in the summer of 2022.

**Figure 3:**
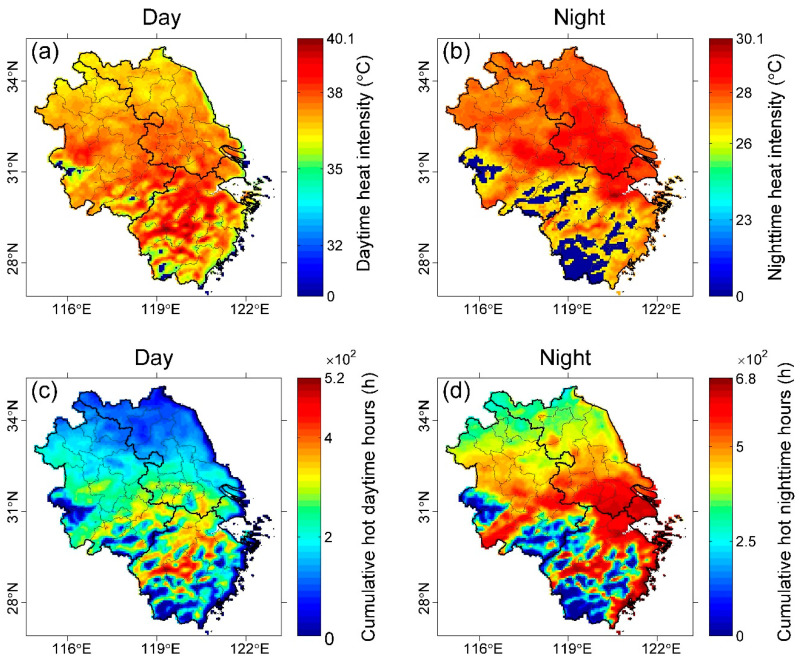
Distribution of the daytime heat intensity (**a**), nighttime heat intensity (**b**), cumulative hot daytime hours (**c**), and cumulative hot nighttime hours (**d**) in the YRD region from July to August 2022. Areas that experienced zero days/hours with *T_max_* ≥ 35 °C or *T_min_* ≥ 26 °C were assigned a value of zero.

To analyze the intensity and spatial distribution of the strongest summer heat, the heat averages on hot days (Figure 3a) and hot nights (Figure 3b) were analyzed. In terms of the daytime heat intensity (Figure 3a), the ratios of the areas in which the average *T_max_* value was ≥35 °C, 38 °C, and 40 °C to the total area of the YRD region were 98.2%, 13.3%, and 0.04%, respectively. The highest average *T_max_* value, 40.2 °C, occurred in Shaoxing, Zhejiang. Compared to other provinces, the daytime heat intensity was strongest in Zhejiang, where 28.7% of the region experienced average *T_max_* values ≥ 38 °C, covering an area of 3.8 × 10^6^ km^2^; this area was 2.3, 10.6, and 114.0 times higher than the corresponding areas in Anhui, Jiangsu, and Shanghai, respectively. Strong daytime heat intensities were observed in most regions, especially Zhejiang.

Regarding the nighttime heat intensity, the ratios of the regions where the average *T_min_* value ≥ 26 °C, 28 °C, and 30 °C to the total YRD area were 89.3%, 26.4%, and 0.02%, respectively (Figure 3b). More areas (10.7%) were classified as non-heat areas during nighttime due to the average *T_min_* value being <26 °C. The highest average *T_min_* value was 30.1 °C, which occurred in Hangzhou, Zhejiang. In contrast to the daytime heat intensity, the nighttime heat intensities in Zhejiang and Anhui were weaker, and only 12.6% and 14.8% of these regions had average *T_min_* values ≥ 28 °C. For Jiangsu and Shanghai, the nighttime heat intensity was stronger, and 53.8% and 67.4% of these regions had average *T_min_* values ≥ 28 °C, covering areas of 7.1 × 10^6^ km^2^ and 7.8 × 10^5^ km^2^, respectively.

Apart from the heat intensity, the heat frequencies during daytime and nighttime were also compared. In terms of the daytime heat frequency, the maximum number of hot daytime hours reached 521 h; this hot-hour duration occurred in Jinhua, Zhejiang. The ratios of the regions in which the hot daytime hours were ≥200 h, 300 h, and 400 h in the entire YRD region were 45.4%, 13.0%, and 1.7%, respectively (Figure 3c). Zhejiang had the strongest daytime heat frequency, with 30.0% of its region experiencing ≥300 hot daytime hours, covering an area of 4.0 × 10^6^ km^2^. In Jiangsu, Anhui, and Shanghai, areas that experienced ≥300 hot daytime hours occupied only 4.9%, 6.7%, and 0.8%, respectively, of the corresponding regions. Similar to the daytime heat intensity, the daytime heat frequency was also strongest in Zhejiang. The areas that experienced ≥400 hot daytime hours were all located in Zhejiang, covering an area of 7.4 × 10^5^ km^2^.

The nighttime heat frequency was more severe than daytime heat frequency, and the ratios of areas within the YRD region where the hot nighttime hours were ≥400 h, 500 h, and 600 h were 58.4%, 31.2%, and 12.4%, respectively (Figure 3d). The maximum cumulative hot nighttime duration reached 682 h, which occurred in Wenzhou, Zhejiang. In contrast to the daytime heat frequency, the nighttime heat frequency was most severe in Shanghai, the whole area of which experienced ≥500 hot nighttime hours. In Zhejiang, Jiangsu, and Anhui, the ratios of the areas that experienced ≥500 hot nighttime hours within the regions were 43.2%, 32.1%, and 17.0%, respectively. Areas that experienced ≥650 hot daytime hours were located in Zhejiang and Shanghai, covering areas of 6.4 × 10^5^ km^2^ and 5.3 × 10^4^ km^2^, respectively.

### 3.2. Strong Heat Risk, Strong Heat Sensitivity and Low Heat Adaptability in Most Areas of the YRD Region

**Figure 4:**
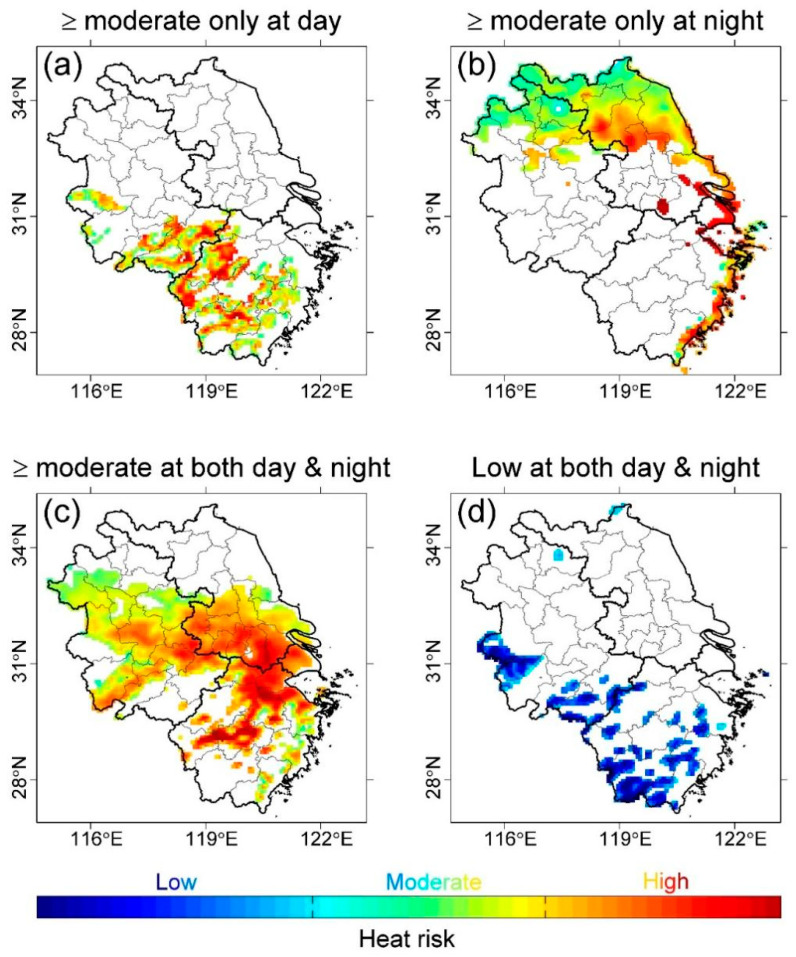
Distribution of areas with heat risks above the moderate level only during daytime (**a**), with heat risks above the moderate level only during nighttime (**b**), with heat risks above the moderate level during both daytime and nighttime (**c**), and with low heat risks during both daytime and nighttime (**d**) over the YRD region.

The natural breaks (Jenks) method [39,40,41], i.e., a method that clusters data into groups that minimize the within-group variance and maximize the between-group variance, was used herein to divide the heat risk into three categories: low, moderate, and high. Areas with low heat risks during both daytime and nighttime account for only 7.5% of the YRD region and were mainly distributed in Jiangsu and Anhui (Figure 4d). These areas within the YRD region with heat risks ≥moderate level only during daytime (representing a daytime heat risk caused by a single factor), with heat risks ≥ moderate level only during nighttime (representing a nighttime heat risk caused by a single factor), and a heat risk ≥ moderate level during both daytime and nighttime (representing a compound heat risk) composed 17.3% (Figure 4a), 15.4% (Figure 4b), and 59.7% (Figure 4c) of the entire YRD region, respectively. Areas with daytime heat risks caused by a single factor were distributed mainly in Zhejiang (60.5%) and Anhui (39.2%). Areas with nighttime heat risks caused by a single factor were distributed mainly in Zhejiang (21.7%), Jiangsu (60.3%), Anhui (13.7%), and Shanghai (4.3%). A total of 12.3% of the YRD region experienced high heat risks during both daytime and nighttime, covering an area of 5.4 × 10^6^ km^2^. These areas were distributed in Zhejiang (39.6%), Jiangsu (32.7%), Anhui (23.5%), and Shanghai (4.2%). Strong compound heat risks were observed in most regions.

**Figure 5:**
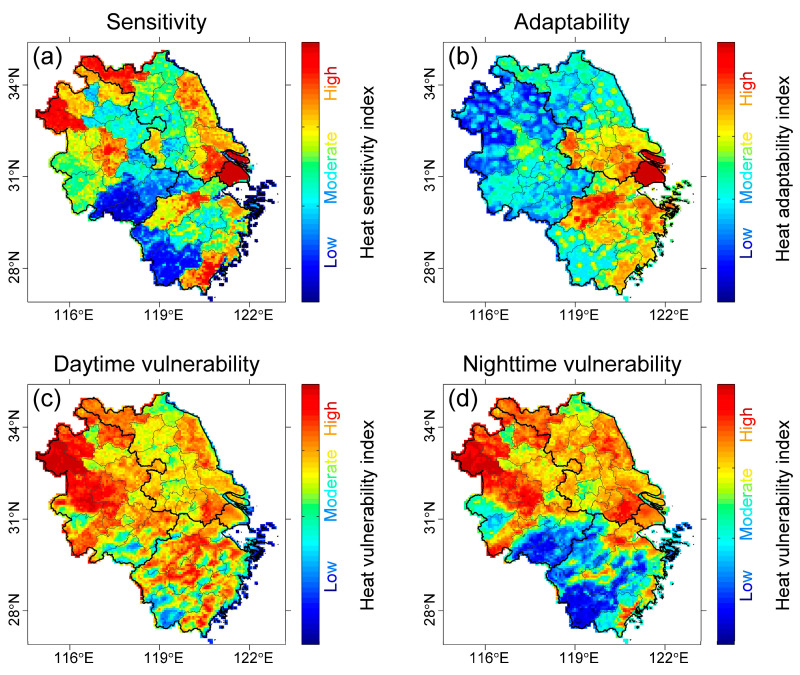
Distributions of the heat sensitivity (**a**), heat adaptability (**b**), daytime heat vulnerability (**c**), and nighttime heat vulnerability (**d**) results in the YRD region.

In addition to the heat risks, the heat sensitivity and heat adaptability results were also divided into three categories: low, moderate, and high. Obvious spatial differences were observed in both indexes. A total of 75.9% of the YRD region experienced heat sensitivity above the moderate level, and these areas were distributed in Zhejiang (24.5%), Jiangsu (35.7%), Anhui (36.7%), and Shanghai (3.0%) (Figure 5a). A total of 31.2% of the YRD region had high heat sensitivity, covering an area of 1.4 × 10^7^ km^2^. Shanghai had the most severe heat sensitivity, with 81.4% of its area experiencing high heat sensitivity. The heat sensitivity conditions were better in Zhejiang and Anhui, with 37.1% and 28.1% of these regions experiencing low heat sensitivity. In contrast, only 6.8% and 10.3% of the Jiangsu and Shanghai regions experienced low heat sensitivity.

Compared to the heat sensitivity results, the spatial differences in heat adaptability were relatively large (Figure 5b), which may be partly related to the difference in where there are hospitals and green space. A total of 73.5% of the YRD region exhibited heat adaptability below the moderate level, and these areas were distributed in Zhejiang (19.6%), Jiangsu (28.1%), and Anhui (52.3%). The whole area of Shanghai exhibited high heat adaptability. Anhui had the weakest heat adaptability; only 1.0% of this region exhibited high adaptability, while 67.2% of the region exhibited low adaptability. The heat adaptability was also weak in Jiangsu, where 70.9% of the area exhibited heat adaptability below the moderate level, most of which was in the northern regions. Apart from Shanghai, Zhejiang exhibited relatively strong heat adaptability; a total of 51.4% of its region presented high heat adaptability, while only 11.9% of the region exhibited low heat adaptability.

### 3.3. Distributions and Causes of Compound Heat Vulnerability in the YRD Region

Based on the heat risk, heat sensitivity, and heat adaptability analysis results, the heat vulnerability was calculated according to the vulnerability assessment framework described above. Only 3.1% of the YRD region displayed low heat vulnerability during both daytime (Figure 5c) and nighttime (Figure 5d). The ratios of the YRD region experiencing heat vulnerability above the moderate level during daytime and nighttime reached 89.4% and 75.6%, respectively. The daytime heat vulnerability was most serious in Anhui, where 56.3% of the region experienced high daytime heat vulnerability, 1.8~2.0 times higher than the area affected for other provinces. The nighttime heat vulnerability was lowest in Zhejiang, where only 12.6% of the region experienced high nighttime heat vulnerability, 4.1~5.7 times lower than the corresponding percentages in other provinces.

Strong heat vulnerability caused by a single factor and multiple factors were both observed in the study area. The areas experiencing strong heat vulnerability caused by a single factor during daytime and nighttime occupied only 16.8% and 3.1% of the YRD region, respectively. A total of 72.6% of the YRD region experienced heat vulnerability above the moderate level during both daytime and nighttime; these areas were distributed mainly in Zhejiang (15.8%), Jiangsu (38.4%), Anhui (43.1%), and Shanghai (2.7%). The compound heat vulnerability was most severe in Anhui, where 45.8% of the area experienced high heat vulnerability during both daytime and nighttime, 1.5~1.7 times greater than the corresponding percentages obtained for Jiangsu and Shanghai. Zhejiang had the lowest compound heat vulnerability; only 8.2% of its region experienced high heat vulnerability during both daytime and nighttime. The ratios of areas that suffered heat vulnerability above the moderate level during both daytime and nighttime in Zhejiang, Jiangsu, Anhui, and Shanghai were 38.9%, 96.0%, 80.8%, and 75.6%, respectively.

**Figure 6:**
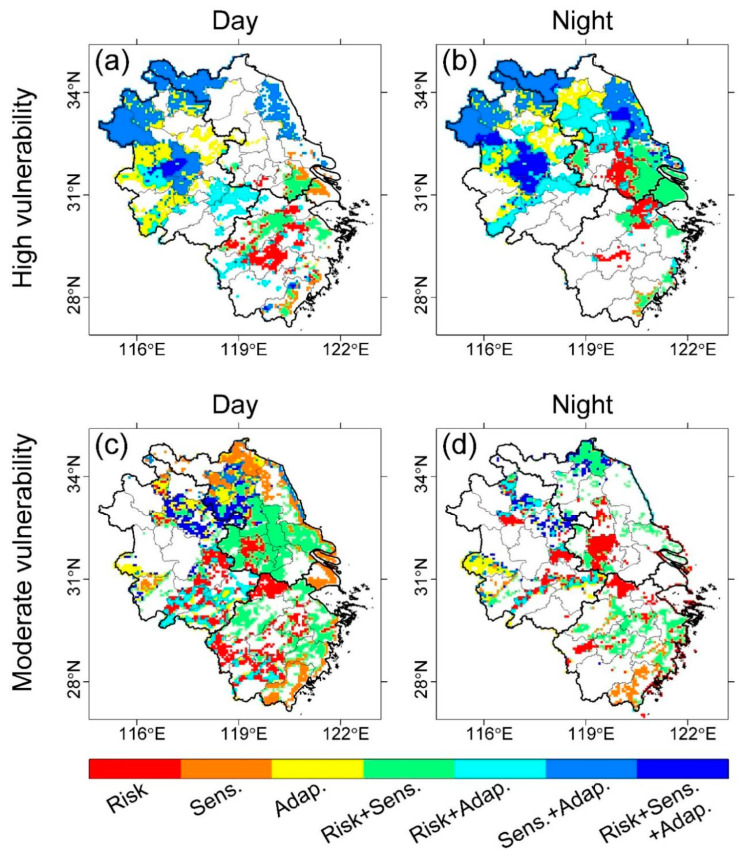
Distributions of areas with different dominant causes of high heat vulnerability during daytime (**a**), high heat vulnerability during nighttime (**b**), moderate heat vulnerability during daytime (**c**), and moderate heat vulnerability during nighttime (**d**) in the YRD region.

Furthermore, the dominant causes of strong heat vulnerability during both daytime (Figure 6a,c) and nighttime (Figure 6b,d) were analyzed. In most areas of the YRD region, heat vulnerability was caused by multiple factors rather than by one single factor. Regarding daytime heat vulnerability, in 67.7% of the total vulnerable areas in the YRD region, daytime heat vulnerability was caused by multiple factors, while areas with a single heat risk/sensitivity/adaptability cause occupied only 13.4%/12.9%/5.9% of the entire region. This phenomenon was more obvious during nighttime; in 79.3% of the total vulnerable areas in the YRD region, nighttime heat vulnerability was caused by multiple factors, and areas with a single heat risk/sensitivity/adaptability cause occupied only 12.0%/4.8%/3.9% of the study area.

For regions with high heat vulnerability, the heat sensitivity + adaptability combination was the largest dominant cause during both daytime (Figure 6a) and nighttime (Figure 6b), impacting 39.1% and 33.6% of the total vulnerable areas in the YRD region, respectively. The heat adaptability and heat risk + adaptability were the following dominant causes affecting vulnerable areas, impacting 19.7% and 14.2% of the YRD, respectively, in daytime and 18.2% and 18.0% of the YRD, respectively, at night. However, for areas with moderate heat vulnerability, the risk + sensitivity combination was the largest dominant cause during both daytime (Figure 6c) and nighttime (Figure 6d), impacting 36.8% and 41.9% of the vulnerable areas, respectively. In daytime, the risk and sensitivity were the next most dominant causes, affecting 17.5% and 15.0% of the vulnerable areas, respectively. For nighttime, the risk and risk + sensitivity + adaptability factors were the next most dominant causes, impacting 24.1% and 10.4% of the vulnerable areas, respectively.

The heat risk and risk + sensitivity were the major causes of heat vulnerability in Zhejiang in daytime, impacting 30.7% and 52.6% of the regional vulnerable areas, respectively. For Jiangsu, the heat sensitivity and risk + sensitivity were the major causes of daytime heat vulnerability, affecting 25.7% and 46.5% of the regional vulnerable areas, respectively. For Anhui, the heat risk + sensitivity and risk + sensitivity + adaptability combinations were the major causes of daytime heat vulnerability, affecting 20.4% and 41.2% of the regional vulnerable areas, respectively. The major causes of heat vulnerability were similar between daytime and nighttime in most regions except Shanghai. In Shanghai, the heat sensitivity was the dominant cause of daytime heat vulnerability, impacting 76.0% of the vulnerable areas, whereas for nighttime, the heat risk + sensitivity combination was the dominant cause, affecting 89.3% of the regional vulnerable areas.

## 4. Discussion

In the summer of 2022, high-pressure systems stagnated and led to long-persistent heat waves in the YRD region. These persistent heat waves caused heat stress that was variably and unevenly distributed across the YRD region because of the demographic and geographic variability across the region. This heat pressure was the comprehensive result of natural and anthropogenic forcings [42]. Among the natural factors, summer heat waves are always accompanied by high-pressure systems, e.g., the Western Pacific subtropical high, and thus by less cloudy and rainy weather [43]. On hot days, more solar radiation can travel through the atmosphere to the land surface due to the lower cloud cover, a larger angle of solar altitude, and other factors [44]. Therefore, during the day, more solar radiation directly or indirectly heats the land surface and atmosphere, causing a rapid rise in *T_a_* [45]. During the day, more solar radiation energy is absorbed and stored in the land surface, and this stored energy releases more longwave radiation and increases the heat conditions throughout the following night [46].

Regarding anthropogenic factors, during the urbanization process, large areas of vegetation and water-body land cover types are replaced by artificial surfaces and buildings with greater heat capacities and thermal conductivities [47]. Urbanization gradually changes the physical properties of the land surface, such as its thermal properties and surface reflectance [48]. In contrast to vegetation surfaces, urban surfaces tend to absorb and store more solar radiation energy during the day and release more longwave radiation at night, thus contributing to the urban heat island effect [49,50,51]. Moreover, the increased amount of heat released from industrial activities, transportation, electrical appliances, and other anthropogenic sources also heats the air during the urbanization process [52,53]. With urbanization comes the loss of green vegetation, especially trees; this means the dual loss of shading and cooling from evapotranspiration. With the rapid urbanization progress and increasing population in the YRD region, the impact of anthropogenic forcings on summertime heat pressure will continuously increase [54,55,56].

In the summer of 2022, *T_max_* and *T_min_* records were continuously broken in many places within the YRD region, and severe heat was observed during both daytime and nighttime. Zhejiang had the strongest daytime heat intensity and heat frequency, and its nighttime heat was also in strong contrast to the extensive, mountainous forested areas in the southwest region. Significantly different from the daytime heat results, nighttime heat was much stronger in densely urban areas than in rural areas; this was attributed to the strong nighttime urban heat island effect. Severe and compound heat risks were observed in the YRD region; only 7.5% of the overall area experienced low heat risks during both daytime and nighttime, and most areas (59.7%) experienced compound heat risks. The high heat sensitivity and low heat adaptability conditions observed in most areas further aggravated the heat vulnerability, causing 72.6% of the YRD region to experience compound heat vulnerability.

Compared to other provinces, although Zhejiang had the most severe daytime heat risk, the higher heat adaptability of this province led to its lower heat vulnerability, especially at night; only 44.5% of this region presented heat vulnerability. Among all of the analyzed provinces, although Jiangsu had the smallest area of severe daytime heat risks, its high heat sensitivity and low heat adaptability led to high heat vulnerability, and 96.3% of Jiangsu exhibited daytime heat vulnerability. For Shanghai, in spite of its small area, its population was much denser and more complex than those of other provinces, and this led to Shanghai having very high heat sensitivity and further intensified the heat vulnerability in this province, especially during nighttime; only 92.6% of this region was identified as heat-vulnerable. Anhui had the weakest heat adaptability, with 67.2% of the region experiencing low heat adaptability. Therefore, the most severe heat vulnerability during both daytime and nighttime occurred in Fuyang, Anhui, despite its lower heat risks compared to other cities.

Although 92.5% of the YRD region was heat-vulnerable, the dominant causes of this heat vulnerability varied. Heat risks caused by a single factor and multiple factors were both observed in each province. Heat risks in most vulnerable areas were caused by multiple factors during both daytime (67.7%) and nighttime (79.3%). It is thus urgent to take efficient, comprehensive measures to address heat vulnerability based on the distributions of its dominant causes. For Zhejiang and Shanghai, projects designed to decrease the urban heat island effect and lower the local heat sensitivity are most important. For Jiangsu and Anhui, measures aiming to decrease the urban heat island effect and improve heat adaptability are most important, especially in the regions of highest vulnerability in Anhui. Detailed, comprehensive measures could be applied in each district and county based on the distributions and causes of heat vulnerability.

## 5. Conclusions

In this study, hourly meteorological data and multisource of socioeconomic data were collected to analyze heat vulnerability during the record-breaking hot summer of 2022 in the YRD region. Severe heat intensities and heat frequencies were observed during both daytime and nighttime. The proportions of areas with daytime heat risks only, nighttime heat risks only, and both heat risks within the entire study area were 17.3%, 15.4%, and 59.7%, respectively. Only 7.5% of the YRD region experienced low heat vulnerability during both daytime and nighttime. The ratios of areas experiencing daytime heat vulnerability only, nighttime heat vulnerability only, and both heat vulnerability were 16.8%, 3.1%, and 72.6%, respectively. The causes of heat vulnerability vary in the YRD region, and the ratios of vulnerable areas caused by multiple factors were 67.7% and 79.3% during daytime and nighttime, respectively. For Zhejiang and Shanghai, projects designed to decrease the urban heat island effect and lower the local heat sensitivity are most important. For Jiangsu and Anhui, measures aiming to decrease the urban heat island effect and improve heat adaptability are most important.

The results indicated severe heat risks in the YRD region during both daytime and nighttime. The high heat sensitivity and low heat adaptability identified in most areas have further aggravated the heat vulnerability. The inhomogeneities in heat sensitivities and heat adaptabilities have further expanded the spatial heterogeneity of heat vulnerability within the YRD, thus impeding the integrated development of the YRD region. These results indicate that detailed, comprehensive measures need to be taken in each district and county to reduce the strong heat vulnerability based on the region-specific dominant causes. This study could help provide scientific support and a reference with which the government can effectively reduce the damages caused by summer heat waves in the YRD region.

## Figures and Tables

**Table 1:** Definitions of hot days, hot nights, hot daytime hours, and hot nighttime hours.

Indicators	Definitions
Hot days	Days when *T_max_* ≥ 35 °C
Hot nights	Nights when *T_min_* ≥ 26 °C
Hot daytime hours	Hours when *T_a_* ≥ 35 °C during daytime
Hot nighttime hours	Hours when *T_a_* ≥ 26 °C during nighttime

**Table 2:** Heat vulnerability assessment framework.

Indexes	Indicators	Weights	Definitions
Daytime heat risk	Heat averages of hot days	0.1954	The average *T_max_* of hot days
	Heat sums of hot days	0.2591	The total number of hot days
	Heat averages of hot daytime hours	0.1731	The average *T_a_* of hot daytime hours
	Heat sums of hot daytime hours	0.3723	The total number of hot daytime hours
Nighttime heat risk	Heat averages of hot nights	0.2615	The average *T_min_* of hot nights
	Heat sums of hot nights	0.3712	The total number of hot nights
	Heat averages of hot nighttime hours	0.2246	The average *T_a_* of hot nighttime hours
	Heat sums of hot nighttime hours	0.1427	The total number of hot nighttime hours
Heat sensitivity	Pop	0.2342	Population density index
	Child	0.2036	Children population (under 12 years old) density index
	Elder	0.2221	Elderly population (over 60 years old) density index
	Jobless	0.2033	Unemployed population density index
	Uneducated	0.1368	The density index of population that have never been educated
Heat adaptability	HCI	0.2381	Hospital coverage index
	PPI	0.2618	Purchasing power index
	TMPI	0.1533	Total medicine products index
	Well-educated	0.2354	Well-educated population (above high school) density index
	Veg	0.1115	Vegetation proportion index

**Table 3:** Definition of different dominant causes of heat factors.

Cause Types	Dominant Causes	Definitions
Single factor	Risk	Heat risk ≥ high-level, heat sensitivity < high-level, heat adaptability > high-level
	Sensitivity	Heat risk < high-level, heat sensitivity ≥ high-level, heat adaptability > high-level
	Adaptability	Heat risk < high-level, heat sensitivity < high-level, heat adaptability ≤ high-level
Multiple factors	Risk + Sensitivity	Heat risk ≥ high-level, heat sensitivity ≥ high-level, heat adaptability > high-level
	Risk + Adaptability	Heat risk ≥ high-level, heat sensitivity < high-level, heat adaptability ≤ high-level
	Sensitivity + Adaptability	Heat risk < high-level, heat sensitivity ≥ high-level, heat adaptability ≤ high-level
	Risk + Sensitivity + Adaptability	Other conditions

The detailed values of “high-level” for daytime heat risk, nighttime heat risk, heat sensitivity, and heat adaptability were 0.5469, 0.5475, 0.5388, and 0.5101, respectively.

## Data Availability

Not applicable.
